# Optical induction of auditory perception via cochlear stimulation in Mongolian gerbils without genetic modification

**DOI:** 10.1016/j.isci.2026.116588

**Published:** 2026-06-30

**Authors:** Yuta Tamai, Miku Uenaka, Aya Okamoto, Yuki Ito, Kaito Fukada, Ayase Kawasaki, Takaki Shintani, Riko Nakagawa, Koji Toda, Shizuko Hiryu, Kohta I. Kobayasi

**Affiliations:** 1 Neuroethology and Bioengineering, Graduate School of Life and Medical Sciences, Doshisha University, Kyotanabe, Kyoto, Japan; 2 Neurobiology of Social Communication, Department of Otolaryngology-Head and Neck Surgery, Hearing Research Centre, University of Tübingen, Medical Center, Elfriede-Aulhorn-Strasse 5, 72076 Tübingen, Germany; 3 Department of Psychology, Keio University, Tokyo, Japan; 4 Faculty of Sociology, Yamato University, Suita, Osaka, Japan

**Keywords:** auditory perception, brain-machine interfaces, classical conditioning, cochlear implants

## Abstract

Whether auditory perception can be non-invasively induced by optical stimulation without genetic modification remains an open question in sensory neuroscience and neuroprostheses. This study presents the first demonstration that transtympanic infrared laser stimulation of the cochlea evokes reliable auditory-guided behavior in awake animals. Mongolian gerbils (*Meriones unguiculatus*) were subjected to classical conditioning where a reward water delivery was predicted by either cochlear laser stimulation or sound stimulus. Laser-conditioned animals successfully learned licking behavior, with conditioned responses and behavioral properties comparable to auditory-conditioned animals. The laser-evoked response was significantly inhibited by auditory masking, and auditory-conditioned animals demonstrated stimulus generalization to laser stimulation. These findings provide behavioral-level evidence that transtympanic infrared laser stimulation can evoke an auditory percept. Our work establishes a foundation for exploring contactless optical stimulation as a non-invasive strategy for engaging the auditory periphery and informs future development of optical approaches to auditory prosthetic technologies.

## Highlights

• Transtympanic laser stimulation evokes auditory-guided learned behavior
• Laser stimulation induces auditory perception in an intensity-dependent manner
• The laser-evoked response is significantly inhibited by auditory masking
• Stimulus generalization to laser stimulation occurs in auditory-conditioned animals

## Graphical abstract

**Figure:**
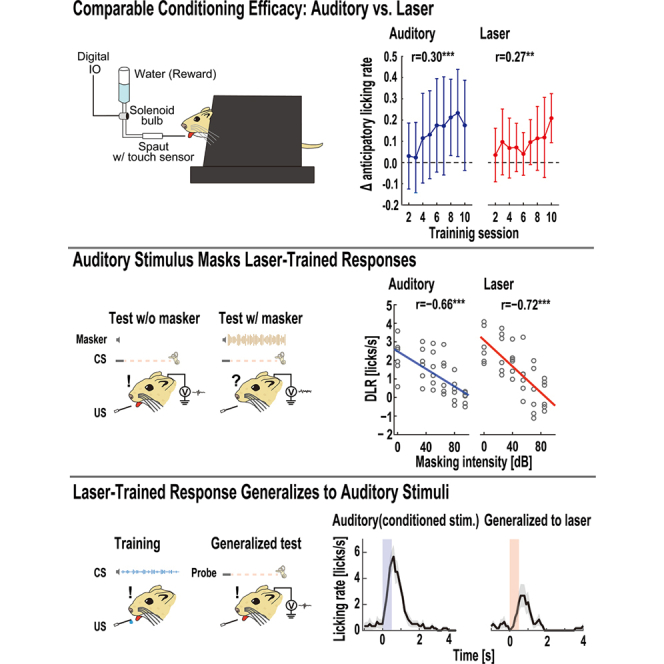

## Teaser

Biological sciences; Neuroscience; Systems neuroscience; Sensory neuroscience; Cognitive neuroscience

## Introduction

Approximately 430 million people, constituting 5% of the world’s total population, suffer from hearing loss, leading to significant communication challenges and social isolation.^1^^,^^2^ For those severely hearing impaired, implantable auditory devices, such as cochlear implants, represent a standard therapeutic option.^3^^,^^4^^,^^5^ However, these devices require surgical intervention, followed by the possibility of surgical complications affecting the performance of the device^4^^,^^6^ and limited spectral resolution caused by electric current spreading.^7^

The last decade has seen many attempts to apply optical neural stimulation to brain-machine interfaces. The method has the potential to activate spatially selective cell populations in a non-contact manner, and several studies have demonstrated successful optical neuromodulation without invasive probe implantation.^8^^,^^9^^,^^10^ However, with respect to cochlear implants, most studies have focused on intracochlear optical stimulation to improve the spectral resolution using infrared laser stimulation^11^^,^^12^^,^^13^ or optogenetic stimulation.^14^^,^^15^ While these studies have provided important insights, they generally rely on invasive surgical access to cochlea. In contrast, the potential to exploit the penetrative properties of infrared laser stimulation to activate the cochlea without direct intracochlear access has received comparatively little attention. In particular, a critical unanswered question remains whether laser stimulation applied through the tympanic membrane can generate a perception that is behaviorally meaningful.

Here, we address this gap by assessing, for the first time, whether transtympanic infrared laser stimulation can evoke an auditory-like perception that animals can use for learning and behavior. Building on our prior research demonstrating that transtympanic laser stimulation can elicit cochlear responses^16^ and activate auditory cortical areas,^17^ we systematically characterized laser-evoked responses using both electrophysiological recordings and behavioral conditioning paradigms in Mongolian gerbils (*Meriones unguiculatus*). Rather than attempting to resolve the debated physiological mechanisms of laser-induced activation, the present study focuses on establishing behavioral proof-of-concept evidence for the perceptual relevance of transtympanic laser stimulation, thereby providing a functional foundation for future mechanistic and translational investigation.

## Results

### Transtympanic laser stimulation elicits auditory perception

**Figure 1:**
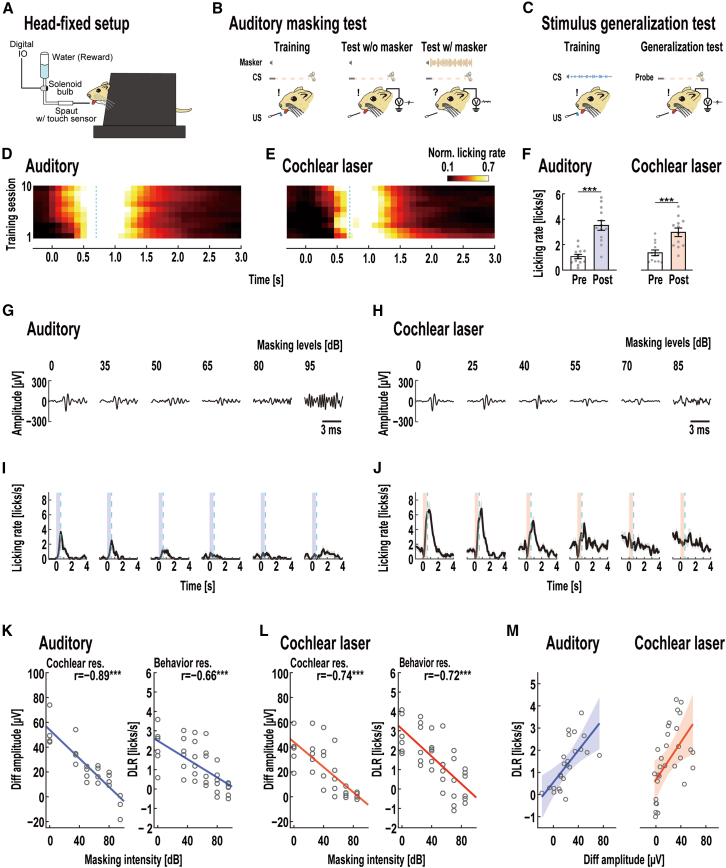
Licking behavior transaction following the training and auditory masking effect on cochlear and behavioral responses evoked by auditory and laser stimulation (A) Schematic demonstration of the head-fixed setup. Experimental workflow of the auditory masking (B) and stimulus generalization (C) are shown (materials and methods for details). Heatmap of the auditory- (D) and laser-induced (E) average peristimulus time histogram (PSTH) of the licking behavior in the rewarded trial of each training session (auditory, *n*=13; laser, *n*=12). Each row represents the mean results for a different training session. One training session comprised 100 trials. The blue dashed lines indicate the reward timing. PSTH was standardized at the peak licking rate in each training session. As the training progressed, the standardized auditory- and laser-induced licking rates were observed earlier by a degree after the CS onset. The color bar indicates licking rate per session, normalized to the peak licking rate. (F) Mean licking rate with auditory (*n*=13) and laser (*n*=12) stimulation on the last training day of catch trials (1–120 trials). The white bars show the mean licking rate for 1 s before stimulus onset. The blue and pink bars indicate the mean licking rate between 0.2 and 1.2 s. The mean licking rates after stimulus onset were significantly higher than those before it in both auditory and laser stimulation. The behavioral response induced by 20 Hz laser stimulation is shown in Figure S3, indicating that licking behavior starts to increase about 100 ms after the stimulus onset. Effect of auditory masking (0, 35, 50, 65, 80, and 95 dB SPL) on CAP from auditory nerves evoked by 80 dB SPL auditory (G) and 13.2 mJ/cm^2^ laser (H) stimulation. Mean PSTH of licking behavior induced by 80-dB SPL auditory (I) and 13.2-mJ/cm^2^ laser (J) stimulation. The blue and red zones in (I) and (J) indicate auditory and laser stimulation periods, respectively. Gray areas show the standard errors of the mean (SEMs) of each trial. Light blue dotted lines represent the expected reward time. Changes in different amplitudes of CAP and DLR induced by auditory (K) and laser (L) stimulation with different masking intensities. As the intensity of the masker on auditory-evoked cochlear and behavioral responses increased, CAP amplitude and DLR significantly decreased (CAP amplitude, r=−0.89, ∗∗∗*p* < 0.001; DLR, r=−0.66, ∗∗∗*p* < 0.001; Pearson’s correlation analysis). A similar tendency was observed in laser-evoked cochlear response and behavioral response, where the intensifying sound pressure level of the masker caused a significant decrease in CAP amplitude (r=−0.74, ∗∗∗*p* < 0.001; Pearson’s correlation analysis) and DLR (r=−0.72, ∗∗∗*p* < 0.001; Pearson’s correlation analysis). (M) Linear regression of DLR changes on the diff amplitudes of CAP. Blue and red lines show straight line fits, and blue and red areas show 95% confidence intervals. As an increase in DLR was observed depending on the different amplitudes of CAP in auditory and laser stimulation conditions (auditory, r=0.80, ∗∗∗*p* < 0.001; laser, r=0.81, ∗∗∗*p* < 0.001; Pearson’s correlation analysis). ANCOVA showed no significant difference in slopes (F[1, 50]=0.177, *p*=0.676; ANCOVA) and *y* axis intercepts (F[1, 51]=0.741, *p*=0.393; ANCOVA) of the auditory- and laser-induced relationship between different amplitudes of peripheral neural response and DLR. (F and K to M) Open gray circles show individual data. Further behavioral alteration by Pavlovian conditioning is shown in supplemental figure (Figure S2).

Mongolian gerbils were trained using a classical conditioning procedure to associate a reward (a drop of water; unconditioned stimulus, US) with either auditory, laser, or visual cues (conditioned stimulus, CS) (Figures 1A–1C; Figure S1; see materials and methods for details). During conditioning, cochlear responses evoked by auditory and laser stimuli were recorded to examine their relationship with behavioral responses. Following training with auditory and laser cues, we further assessed the effects of auditory masking on laser-evoked behavior, examined the dependence of the behavioral responses on laser intensity, and tested stimulus generalization from auditory to laser stimulation. Animals were successfully conditioned using cochlear laser stimulation as the CS (Figures 1D–1F; Figure S2). After approximately 1,000 training trials (10 training sessions), all auditory- and laser-trained animals (auditory, *n*=13; laser, *n*=12) reliably associated the CS with the US. In both groups, anticipatory licking behavior emerged prior to US onset and became more pronounced with continued training. Learning curves for auditory- and laser-conditioned animals showed comparable temporal profiles (Figures 1D and 1E). Consistently, the delta anticipatory licking rate increased progressively over the course of training in both groups (Figure S2; auditory, r = −0.30, *p* < 0.001; laser, r = −0.27, *p* < 0.01; Pearson’s correlation analysis). An analysis of covariance (ANCOVA) revealed no significant difference in the regression slopes describing changes in delta anticipatory licking rate between the auditory- and laser-conditioned groups, indicating similar learning dynamics. However, the intercept was significantly higher in the auditory-conditioned group, indicating a higher overall anticipatory licking rate than in the laser-conditioned group. Together, these results indicate that while auditory conditioning elicited stronger overall behavioral responses, laser stimulation supported a comparable rate of learning during classical conditioning.

Additionally, white noise systematically masked both laser-evoked and auditory-evoked behavioral responses but not visually evoked behavioral response (Figures 1B and 1G–1M; Figure S4). Previous studies revealed that laser-evoked electrophysiological responses can be masked by acoustic stimulation, indicating that laser-evoked responses are processed in peripheral auditory pathways.^18^^,^^19^ The present study extends these findings by directly demonstrating that auditory masking also attenuates laser-evoked behavioral responses, linking peripheral auditory activation to perceptually relevant behavior. The previous study by Matic et al.^12^ investigated laser-induced perceptual events in cats and demonstrated that laser irradiation of the cochlea increased the probability of the animal turning its head to the irradiated side. However, because this behavior was spontaneous and not associated with a learned associative contingency, it did not allow conclusions regarding the perceptual quality or modality of the laser-evoked sensation. In contrast, by employing a classical conditioning paradigm, the present study demonstrates that laser stimulation can serve as an effective CS that supports anticipatory behavior and is subject to auditory masking. These results provide behavioral evidence that laser stimulation of the cochlea can evoke an auditory-like percept, while avoiding stronger claims regarding its precise sensory content.

### Auditory perception can be manipulated by changing radiant energy

**Figure 2:**
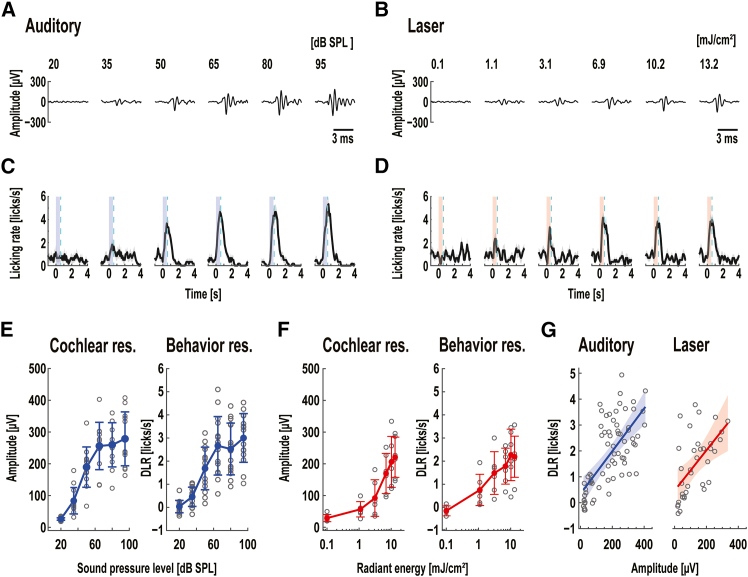
Intensity dependence of cochlear and behavioral responses elicited by auditory and laser stimulation CAP of auditory nerves with auditory (A) and laser (B) stimulation. Mean PSTH of licking behavior elicited by auditory (C) and laser (D) stimulation. In (C) and (D), the blue and red zones indicate auditory and laser stimulation periods, respectively. Gray areas show the SEM for each trial. Light blue dotted lines represent the reward timing in a training session. Increasing the intensity of auditory and laser stimuli produced a systematic change in CAPs and mean PSTHs. Average amplitude of CAP and DLR with auditory (E) and laser (F) stimulation. Error bars indicate standard deviations (SDs). As the sound pressure level increased from 20 to 95 dB SPL, the average CAP amplitude and DLR increased from 26.14 ± 3.87 to 278.63 ± 84.83 (*n*=12; Mean ± SD) and 0.03 ± 0.27 to 3.00 ± 1.05 (*n*=15), respectively. With increasing radiant energy from 0.1 to 13.2 mJ/cm^2^, there was a steady increase in CAP amplitude and DLR (CAP amplitude, from 28.92 ± 11.34 to 220.71 ± 63.38, *n*=6; DLR, from −0.15 ± 0.21 to 2.19 ± 0.89, *n*=6). (G) Correlation between CAP amplitude and DLR elicited by auditory and laser stimulation. Blue and red lines depict straight line fits, and blue and red areas show 95% confidence intervals. Increasing CAP amplitude resulted in a systematic increase in DLR under auditory and laser stimulation (auditory, r=0.80, ∗∗∗*p* < 0.001; laser, r=0.81, ∗∗∗*p* < 0.001; Pearson’s correlation analysis). The slopes and *y* axis intercepts of the fitted linear regression were not statistically different from each other (slope, F[1, 104]=0.046, *p*=0.832; intercepts, F[1, 105]=0.093, *p*=0.761; ANCOVA). (E–G) Open gray circles show individual data. Further intensity dependence of behavioral responses is described in supplemental figure (Figure S5).

The amplitude of the laser-evoked compound action potential (CAP) was well-correlated with the strength of the conditioned response (Figure 2). Although some studies have attempted to show the possibility of manipulating perception in terms of intensity dependency^11^^,^^20^ and spectral selectivity^21^^,^^22^ by comparing auditory- and laser-evoked physiological responses, no studies have ever revealed the relationship between laser-evoked physiological responses and behavioral consequences. Our data showed that both laser-evoked CAP and behavioral responses increased depending on the radiant energy, which were comparable to those on the sound pressure level (Figures 2A–2G). The same was true in the masking experiment (Figures 1K–1M). These suggest that the laser-evoked behavioral responses were mediated by cochlear activation, and laser-evoked perception can be controlled by changing radiant energy, possibly in a similar way to the sound pressure level of auditory stimulation. Importantly, these results establish a direct relationship between laser-evoked cochlear responses and behavioral output, without making assumptions about the precise underlying biophysical mechanism.

### Laser-induced perception is similar to auditory perception elicited by clicking sound, but at least partially different

**Figure 3:**
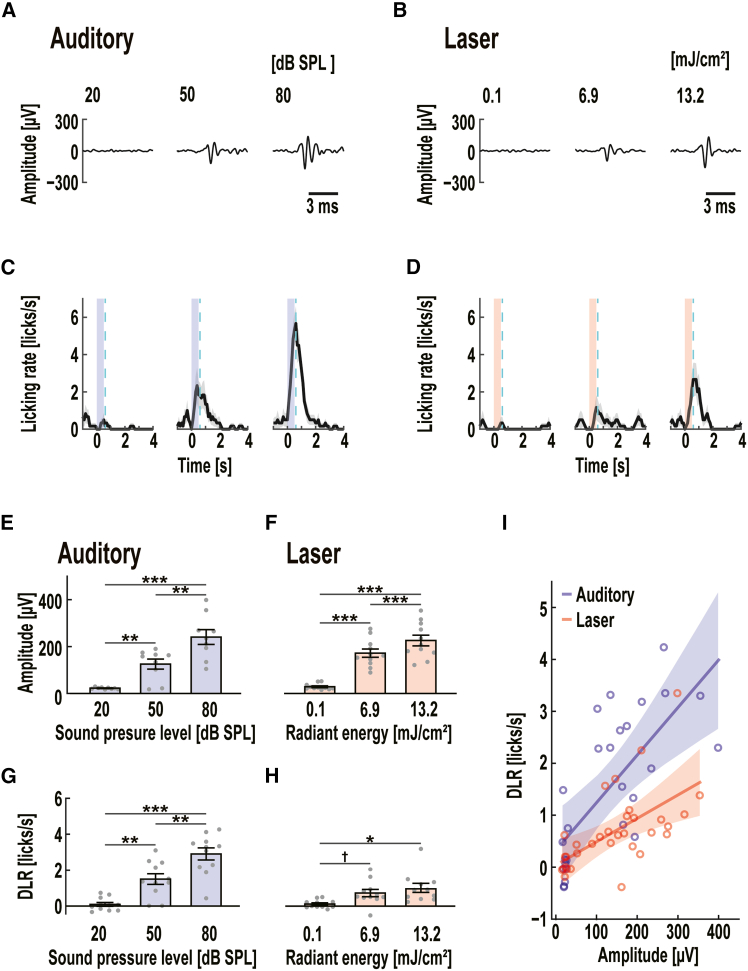
Stimulus generalization from auditory stimulation to laser stimulation CAP from auditory nerves (A and B) and mean PSTH of licking behavior (C and D) elicited by auditory (20, 50, and 80 dB SPL) and laser (0.1, 6.9, and 13.2 mJ/cm^2^) stimulation in subjects classically conditioned with auditory stimulus as CS. The blue and red zones in (C) and (D) indicate auditory and laser stimulation periods, respectively. Gray areas show the SEMs of each trial. Light blue dotted lines represent the reward timing subjects expected. Mean CAP amplitude (E and F) and DLR (G and H) with auditory and laser stimulation. Error bars represent SEMs. Open gray circles show individual data. Auditory- and laser-induced CAP amplitudes significantly increased with stimulus intensity (auditory, 20 dB SPL: 22.45 ± 1.37 μV, 50 dB SPL: 125.33 ± 21.56 μV, 80 dB SPL: 240.79 ± 31.71 μV, *n*=9; laser, 0.1 mJ/cm^2^: 28.74 ± 3.79 μV, 6.9 mJ/cm^2^: 172.04 ± 17.61 μV, 13.2 mJ/cm^2^: 225.83 ± 22.90 μV, *n*=11; Mean±SEM). Laser stimulation induced licking behaviors in auditory-trained animals in an intensity-dependent manner similar to auditory stimulation. As the sound pressure level increased from 20 to 80 dB SPL, DLR increased from 0.09 ± 0.11 to 2.90 ± 0.34 licks/s (*n*=11). Laser-induced DLR gradually changed depending on radiant energy (0.1 mJ/cm^2^: 0.12 ± 0.07 licks/s, 6.9 mJ/cm^2^: 0.73 ± 0.20 licks/s, 13.2 mJ/cm^2^: 1.01 ± 0.25 licks/s, *n*=11). In (E to H), there was a significant difference in response to each stimulus intensity based on two-tailed paired *t* test with Bonferroni correction for multiple comparisons (†*p* < 0.1, ∗*p* < 0.05, ∗∗*p* < 0.01, ∗∗∗*p* < 0.001). (I) DLR as a function of CAP amplitude induced by auditory and laser stimulation. Blue and red lines and shaded areas describe fitted linear regressions and 95% confidence intervals. Open blue and red circles show individual data. The correlation coefficients between CAP amplitude and DLR were statistically significant in both auditory and laser stimulation conditions (auditory, r=0.80, ∗∗∗*p* < 0.001; laser, r=0.81, ∗∗∗*p* < 0.001; Pearson’s correlation analysis). Comparing the linear regression model between auditory and laser stimulation conditions revealed that the difference in slopes and *y* axis intercepts was statistically significant (slopes, F[1, 56]=5.474, ∗*p* < 0.05; *y* axis intercepts, F[1, 57]=18.256, ∗∗∗*p* < 0.001; ANCOVA).

To further examine the behavioral relevance of laser-evoked stimulation, we assessed stimulus generalization from auditory to laser stimulation (Figures 1C and 3). Animals that were conditioned exclusively with auditory stimuli reliably exhibited conditioned responses during probe trials in which laser stimulation was presented, despite having no prior experience with laser stimulation during training (Figures 3A–3H). This finding indicates that transtympanic laser stimulation engages perceptual features that overlap with those evoked by acoustic stimulation. In addition, the magnitude of the generalized behavioral response increased with higher radiant energy of the laser stimulus, indicating that the strength of laser-evoked cochlear activation influences behavioral output. Together, these results demonstrate that laser stimulation of the cochlea can evoke a percept that generalizes from learned auditory cues and that its behavioral impact can be parametrically modulated by radiant energy, without implying that laser- and sound-evoked percepts are identical.

However, the detailed features of auditory perception elicited by transtympanic laser stimulation remain unclear. ANCOVA revealed that the laser-generated linear regression slope in the dependence of delta licking rate (DLR) on CAP in acoustically trained subjects was significantly smaller than the auditory-generated linear regression slope (Figure 3I). Conversely, there was no difference between auditory- and laser-generated dependence in subjects trained on each stimulus modality (Figure 2G). These results indicate that, for a given level of cochlear activation, laser stimulation elicited a weaker conditioned response than auditory stimulation in acoustically trained animals, whereas laser-evoked cochlear responses were nevertheless sufficient to support robust conditioned behavior when used as the training stimulus. This pattern is consistent with established principles of stimulus generalization, in which the conditioned response is maximal for the trained CS and gradually decreases as the perceptual similarity between CS and test stimuli diminishes.^23^^,^^24^ Accordingly, the present findings suggest that pulsed laser irradiation evokes an auditory-like percept that overlaps with, but is not identical to, the percept evoked by the acoustic click train used during training. Supporting this interpretation, tonal masking at 1.4, 2.8, and 5.6 kHz significantly attenuated laser-evoked cochlear responses (Figure S6), indicating that laser stimulation engages frequency-dependent auditory processing that differs from the broadband characteristics of click stimuli. Future studies using laser-trained animals and systematic transfer tests to auditory stimuli with defined spectral properties will be required to more precisely characterize the perceptual content evoked by transtympanic laser stimulation.

## Discussion

### The effect of optoacoustic phenomena in laser-induced auditory response

The mechanism by which cochlear laser stimulation evokes neural and behavioral responses is still largely unresolved. Following the initial study by Wells et al.,^25^ which demonstrated that laser irradiation of sciatic nerves yielded CAP *in vivo*, subsequent studies delved into the underlying mechanism of the laser-evoked neural response. These studies suggest that the optical absorption of water causes an instantaneous temperature rise in cells,^26^^,^^27^^,^^28^ resulting in reversible alterations in electric capacitance of the plasma membrane^29^ or changes in heat-induced ion channel activity.^30^ The thermal build-up in cochlear cells also results in basilar membrane vibration with lymph expansion, causing hair cell depolarization (optoacoustic effect).^31^^,^^32^^,^^33^^,^^34^ Teudt et al.^31^ also showed that pulsed infrared laser irradiation with radiant energies below 13.2 mJ/cm^2^ could generate airborne sound pressures of approximately 35–40 dB SPL. However, in our experiments, no cochlear responses were detected when laser irradiation was directed away from the cochlea, indicating that simple airborne sound generation alone is unlikely to account for the observed effect under our experimental conditions (Figure S7). Xia et al.^33^ reported that laser stimulation at 13.9–182.3 mJ/cm^2^ might induce basilar membrane vibration corresponding to sound between 46 and 74 dB SPL, and the sound level could be 53.5 dB SPL when laser pulses at 18.9 mJ/cm^2^ (threshold radiant energy) were irradiated to the cochlea. Although our results are not directly comparable to these data due to differences in laser parameters (e.g., spot size, laser irradiation time, and repetition rate), which are challenging to accurately determine in an *in vivo* experiment, as mentioned by Tan et al.,^35^ and tissue parameters (e.g., absorption coefficients, density, and thermal conductivity), these results suggest that our laser stimulation potentially induced nonnegligible lymph vibration in the cochlea. Additionally, some studies have shown direct activation of electrophysiological responses in hair cells^36^^,^^37^^,^^38^; therefore, laser stimulation has the sufficient possibility of inducing hair cell depolarization.

To explore hair cell function in laser-induced cochlear activation, several studies^34^^,^^39^^,^^40^ compared laser-evoked auditory responses in normal hearing and chemically deteriorated cochlea. They demonstrated that laser-evoked cochlear responses were absent in chemically impaired cochlea, suggesting mediation by hair cells. In contrast, Richter and colleagues^41^ reported observing the cochlear response induced by laser stimulation after a 30–40 dB chemical deafening of the auditory threshold. They noted that the laser-induced response could not be masked by white noise in animals with chemically deteriorated cochleae,^19^ suggesting that the contribution of mechanical and non-mechanical components may vary depending on stimulation conditions and cochlear integrity. In this context, our observation that the reduction in response amplitude was more pronounced for auditory-evoked potentials than for laser-evoked potentials (Figure S8) is consistent with previous reports, suggesting a partial dissociation between auditory- and laser-induced cochlear activation. Tan et al.^42^ showed that laser stimulation could induce CAP in congenitally hearing-impaired mice with a synaptic transmission deficiency between inner hair cells and spiral ganglion neurons. Xia et al.^33^ reported a higher neural response when the laser beam targeted spiral ganglion neurons compared to the cochlear wall, while cochlear pressure remained almost independent of the laser irradiation site.^33^^,^^35^ These findings suggest that laser stimulation bypasses the need for hair cell depolarization, directly eliciting a cochlear nerve response. Teudt et al.^31^ reported that only CAPs were observed when the laser fiber was positioned 2 mm away from the cochlea, whereas both cochlear microphonics (CM) and CAP were observed when the fiber was placed 1 mm from the cochlea. This finding is consistent with our results, in which both auditory-induced CM and CAP were clearly recorded, whereas a repetitive pulsed laser train from the outer ear induced only CAPs, as reported in previous studies, including ours^16^^,^^17^^,^^20^ (Figure S9). Importantly, the present study does not aim to resolve the precise biophysical mechanism underlying laser-induced activation. Rather, our primary contribution is the demonstration that transtympanic laser stimulation can generate a percept that animals can use behaviorally. Our stimulus generalization results indicate that laser stimulation produces a percept that is similar to, but not identical with, that evoked by broadband click sounds (Figure 3I). Such partial generalization is consistent with differences in stimulus features within a shared perceptual space and does not, by itself, specify the underlying mechanism. While tonotopic activation patterns in the inferior colliculus have been reported to resemble those elicited by acoustic clicks under certain laser stimulation conditions,^34^ the relationship between peripheral activation mechanisms and perceptual outcomes remains to be clarified. Future studies are essential to investigate the physiological mechanism of laser-evoked auditory perception by comparing the effects of laser irradiation at different degrees and types of peripheral hearing impairment and by combining behavioral assays with refined biophysical measurements. Establishing such mechanistic specificity will be essential for evaluating the translational potential and limitations of cochlear laser stimulation. The present findings provide a functional, behaviorally grounded foundation for these future investigations.

### Clinical utility of laser stimulation for conductive hearing loss

Even if the laser-evoked CAP is an optoacoustically induced response mediated by hair cells rather than by direct stimulation of the cochlear nerve, several studies have explored the clinical utility of laser stimulation. Baumhoff et al.^34^ demonstrated that the frequency characteristics of the pressure wave induced by intracochlear optoacoustic stimulation correspond to the tonotopic neural response in the inferior colliculus. Additionally, the optoacoustically induced pressure waves in the cochlea were shown to be sufficiently intense even in the human cochlea.^43^ These findings suggest that intracochlear optoacoustic stimulation could potentially be combined with electrical cochlear implants as an “optical actuator.” A series of studies by Wenzel e.g.,^44^^,^^45^^,^^46^ have investigated optoacoustic stimulation of the tympanic membrane, demonstrating that the optoacoustic stimulation can control tympanic membrane vibrations and elicit auditory brainstem responses. These results suggest that optoacoustic stimulation may represent a possible novel approach to reduce discomfort caused by acoustic feedback in hearing aids.

The present study suggests that transtympanic laser stimulation may retain potential advantages as a middle-ear-based stimulation strategy, even if laser-evoked auditory responses are mediated primarily through optoacoustic activation of cochlear hair cells. Middle ear implants are typically indicated for patients with moderate to severe hearing loss who derive limited benefit from conventional hearing aids.^47^ While such implants can deliver enhanced vibrational energy to cochlear hair cells,^48^^,^^49^ their clinical use requires surgical implantation and is associated with risks and complications comparable to those of cochlear implants. Recent optical studies have demonstrated that the long wavelength infrared laser light (e.g., λ=1,871 nm), as used in the present study, is capable of penetrating the tympanic membrane.^50^ Consistent with this property, our previous work showed that laser irradiation delivered from the outer ear can evoke cochlear and auditory cortical responses without mechanical coupling to the ossicular chain.^16^^,^^17^ Extending these observations, we found that CAPs evoked by transtympanic laser stimulation were comparable in magnitude to those elicited by laser stimulation applied directly to the round window membrane, a configuration commonly used in intracochlear optical stimulation studies^18^^,^^20^^,^^51^ (Figure S10). Together, these findings indicate that laser irradiation delivered from the outer ear can activate the cochlea and modulate auditory-related behavior without relying on conventional middle-ear mechanics. While the present data do not establish superiority over existing implantable devices, they suggest that transtympanic laser stimulation could represent a conceptually distinct, non-contact approach for cochlear stimulation within the framework of middle-ear-based auditory interventions. Future studies examining transtympanic laser responses in animal models of conductive hearing loss, such as tympanic membrane perforation or ossicular disruption, will be necessary to evaluate the feasibility and potential clinical relevance of this approach.

### The effect of white noise distraction in behavioral response

The effect of the white noise distraction in behavioral response needs to be carefully considered. Several studies e.g.,^52^^,^^53^^,^^54^ have shown that masking sound can act as a distractor, reducing behavioral responses, in addition to their masking effect on auditory perception. This indicates that the reduction in performance under white noise might not absolutely mean that cochlear laser stimulation induced a behavioral response via the auditory system. Previous studies^55^^,^^56^^,^^57^ have also reported that interference from distractors increases with task difficulty. Therefore, the laser-evoked behavioral response may be more sensitive to the distractor than auditory and visual responses, particularly when behavioral training with laser stimulation is more difficult than with these stimulations. In our study, visual stimulation required the longest training period (mean ± SD training days; auditory, 10.0 ± 3.5, *n*=13; laser, 13.3 ± 3.4, *n*=12; visual, 30.8 ± 11.8, *n*=4), indicating that training with visual stimulation was the most challenging task in the current experiment. Therefore, masking noise can more strongly interfere with the behavior induced by visual stimulation than with that elicited by auditory or laser stimulation. However, our data showed that the masking effect on the visually evoked conditioned response was not significant (r = −0.17, *p* = 0.310), suggesting that the impact of the white noise distraction on conditioned response to auditory and laser stimulation may be less than indicated by the results (Figure S4). In contrast, the auditory- and laser-evoked conditioned responses were significantly reduced by white noise (auditory, r=−0.66, *p* < 0.001; laser, r=−0.72, *p* < 0.001), suggesting that laser stimulation could elicit behavioral responses via the auditory system, although the effect of white noise distraction cannot be fully excluded.

### Overall implications

In summary, this study investigated the feasibility of using infrared laser as a contactless approach to explore alternatives to surgically invasive auditory prosthetic strategies. While previous work has primarily focused on intracochlear optical stimulation to improve spectral resolution using infrared laser stimulation^11^^,^^12^^,^^13^ or optogenetic stimulation,^14^^,^^15^ the potential of transtympanic, contactless laser stimulation has largely been unexplored.^16^^,^^17^ Here, we provide the first behavioral evidence that laser irradiation of the cochlea delivered from the outer ear can elicit a percept that animals can reliably use in a learning task. Animals conditioned with cochlear laser stimulation acquired robust licking responses with behavioral characteristics comparable to those trained with acoustic stimuli. Furthermore, laser-evoked responses were significantly suppressed by auditory masking, and animals trained with sound stimuli exhibited stimulus generalization to laser stimulation, supporting the notion that cochlear laser stimulation engages auditory-related perceptual processes. Although the precise physiological mechanisms underlying laser-evoked auditory perception remain unresolved, our findings establish a behavioral proof-of-concept demonstrating that contactless cochlear laser stimulation can produce functionally meaningful percepts. These results provide an experimental foundation for future studies aimed at clarifying the underlying mechanisms and assessing the potential and limitations of infrared laser stimulation as a non-invasive strategy for auditory modulation.

### Limitations of the study

The first limitation of this research is that the method for tonotopically specific activation of the cochlear nerve was not addressed. In the current research, flat-cleaved optical fiber was used, and the beam width at the cochlea was relatively wide (2.2 mm); the laser stimulus activated a wide frequency region of the cochlea, allowing the validation of the concept of the transtympanic laser stimulation. Therefore, the design of this study did not include achieving spatial selectivity in the cochlea, which helps the laser stimulation to induce frequency-specific activation of the cochlea. Ren et al. controlled the laser focal spot on the cochlea with a precision of 50 μm using a collimating lens. Future studies involving a similar lens system could permit laser stimulation to reconstruct the cochlear tonotopic organization by minimizing the irradiation spot on the cochlea.

Another important limitation of the present study is that it does not fully address the long-term safety and chronic tolerability of transtympanic infrared laser stimulation. While our previous work demonstrated that prolonged transtympanic pulsed laser stimulation at power densities up to 6.6 W/cm^2^ did not induce detectable thermal elevation or cause histological, electrophysiological, or behavioral abnormalities in Mongolian gerbils,^58^ these findings primarily establish the absence of acute or subacute tissue damage under controlled experimental conditions. In the present study, laser-evoked behavioral responses were reliably observed at radiant energies of approximately 1.1 mJ/cm^2^ or higher, corresponding to an average power density of approximately 2.2 W/cm^2^ (repetition rate, 4 kHz; pulse train duration: 500 ms). This indicates that laser-evoked auditory perception can be generated within the previously defined safety range. However, auditory prosthetic devices are intended for repeated use over long periods, potentially spanning years, even if stimulation is applied intermittently rather than continuous. Therefore, the current data do not exclude the possibility of cumulative thermal effects, delayed tissue changes, or subtle functional alterations that may emerge with chronic exposure. Comprehensive long-term safety studies, including extended-duration stimulation paradigms, detailed histopathological analyses, and longitudinal functional assessments, will be essential before transtympanic laser stimulation can be considered for clinical translation. Addressing these issues represents a critical next step in evaluating the feasibility of non-invasive optical approaches for auditory prostheses.

A further limitation is that a detailed histological assessment of the tympanic membrane following transtympanic laser stimulation was not systematically performed. Our previous study showed that visual inspection of the tympanic membrane did not reveal observable trauma, such as perforation or burn marks, following laser irradiation.^59^ These observations are consistent with the notion that laser stimulation within this range does not cause overt tissue damage. However, prior *in vivo*^45^ and *in vitro*^60^ studies assessing the biocompatibility of laser irradiation of the tympanic membrane have reported structural alterations, including subtle tissue damage, even in cases where no substantial shifts in auditory thresholds were observed. Taken together, these findings suggest that transtympanic laser stimulation may induce minor or subclinical irritation or microstructural changes in the tympanic membrane that are not detectable by gross inspection alone, even when the stimulation parameters fall within an established safety margin. Therefore, further studies should incorporate systematic post-stimulation histological analyses of the tympanic membrane to rigorously evaluate potential tissue alterations and to further define operational limits for transtympanic laser stimulation. The present study does not resolve the ongoing controversy regarding the feasibility of laser stimulation as an auditory intervention under conditions of hearing impairment. A critical issue for the clinical applicability of laser-based approach is whether laser irradiation can elicit functionally meaningful auditory response in cases of sensorineural hearing loss, rather than being effective only in conductive hearing loss or in ears with preserved hair cell function. Previous studies addressing this question have yielded inconsistent and sometimes conflicting results. Several investigations comparing laser-evoked auditory responses between normal-hearing animals and chemically deteriorated cochleae have reported an absence of laser-evoked auditory brainstem responses following hair cell damage (Figure S8), suggesting that laser-evoked responses in these paradigms are predominantly mediated by hair cell activation via optoacoustic pressure waves in the cochlear fluid. Under this interpretation, laser stimulation would not be expected to compensate for sensorineural hearing loss. In contrast, studies by Richter and colleagues reported measurable laser-evoked cochlear responses even after chemical deafening in rodents.^41^ These authors further noted that the discrepancies across studies are difficult to interpret, as extensive hair cell loss itself can severely compromise neural function, confounding conclusions regarding the site and mechanism of laser-induced activation.^61^^,^^62^ Given these unresolved inconsistencies, the present behavioral and electrophysiological findings in normal-hearing animals should be interpreted as a proof-of-concept rather than as evidence for clinical efficacy in sensorineural hearing loss. Future studies will require systematic comparisons across multiple models of hearing impairment, including genetically modified animals with well-defined deficits in hair cell or synaptic function, as demonstrated in recent work.^42^ Such approaches will be essential to clarify the physiological mechanisms underlying laser-evoked auditory responses and to rigorously evaluate the feasibility of laser-based auditory prosthetic strategies.

## Resource availability

### Lead contact

Further information and requests for resources should be directed to and will be fulfilled by the lead contact, Yuta Tamai (ytamai@mail.doshisha.ac.jp).

### Materials availability

This study did not generate new unique reagents.

### Data and code availability

All data needed to evaluate the conclusions are shown in the study and supplemental figures. The datasets of this study are available at Mendeley Data (https://doi.org/10.17632/wtg24zbst6.1) and are publicly available. This study does not report original code. Additional data related to this study are available from the corresponding author upon reasonable request.

## Acknowledgments

We would like to thank Hiroshi Riquimaroux for his support during the early stages of this work. We also thank Steffen Hage for technical support and valuable discussion and Yee Ping Cheung and Enago (https://www.enago.com/) for English language editing. Funding: This research was financially supported by the 10.13039/501100001691Japan Society for the Promotion of Science (10.13039/501100001691JSPS) 10.13039/501100001691KAKENHI Grants Nos. 25K21230 (Y.T.), 24KK0210 (Y.T.), 24KJ1927 (Y.T.), 21K21322 (Y.T.), 24H00729 (K.I.K.), 21H03469 (K.I.K.), the 10.13039/501100001691JSPS Overseas Research Fellowship (Y.T.), and Keio Academic Development Fund (K.T.).

## Author contributions

Conceptualization, Y.T. and K.I.K.; data curation, Y.T., M.U., A.O., K.F., A.K., T.S., and R.N.; formal analysis, Y.T. and M.U.; funding acquisition, Y.T., K.T., and K.I.K.; investigation, Y.T. and M.U.; methodology, Y.I., K.T., and S.H.; project administration, Y.T., K.T., S.H., and K.I.K.; software, Y.T. and M.U.; supervision, Y.T., K.T., S.H., and K.I.K.; validation, Y.T., M.U, and A.O.; visualization, Y.T.; writing – original draft, Y.T. and M.U.; writing – review and editing, Y.T., K.T., S.H., and K.I.K.

## Declaration of interests

The authors have no competing interest to declare.

## STAR★Methods

### Key resources table

**Table:** 

REAGENT or RESOURCE	SOURCE	IDENTIFIER
**Deposited data**		

Data and Code for Analyses	Mendeley Data	https://doi.org/10.17632/wtg24zbst6.1

**Experimental models: Organisms/strains**		

Mongolian gerbils (*Meriones unguiculatus*)	SHIMIZU Laboratory Supplies Co., Ltd., Kyoto, Japan, and Graduate School of Life and Medical Sciences, Doshisha University, Kyoto, Japan	N/A

**Software and algorithms**		

MATLAB	MathWorks	RRID: SCR_001622
R	R core team	RRID: SCR_001905
Python	Python Software Foundation	RRID: SCR_008394

### Experimental model and study participant details

#### Animals

In total, thirty-one experimentally naive adult Mongolian gerbils (ten females, twenty-one males; Meriones unguiculatus) aged 2–9 months were utilized. Each gerbil was housed with two to four others in a cage measuring 20 cm (W) × 40 cm (L) × 17 cm (H), with free access to food and water. The animal room maintained a temperature between 22°C and 23 °C, with approximately 50% relative humidity and a 12-h light: 12-h dark cycle. All experimental procedures adhered to the guidelines established by the Ethics Review Committee of Doshisha University. The Animal Experimental Committee of Doshisha University approved the experimental protocols (reference number: A22071).

### Method details

#### Animal surgery

All surgical procedures were conducted under anesthesia using an intramuscular combination injection of ketamine (47.0 mg/kg) and xylazine (9.3 mg/kg). Breathing pattern was monitored throughout the surgery to assess the depth of the anesthesia. Maintenance doses of ketamine (17.5 mg/kg) and xylazine (7.0 mg/kg) were administered every 30–50 min or if the animal exhibited faster respiration rates. Gerbil body temperature was maintained using heating pads positioned beneath the animals.

The parietal and temporal sides of the subject’s head were exposed after shaving the fur. For perioperative analgesia, a topical local anesthetic (xylocaine gel; Aspen Japan, Tokyo, Japan) was applied prior to incision of the skin and removal of the temporal muscle on both sides.

The skull was meticulously cleaned with a 0.3% sodium hypochlorite solution and saline. A small hole was made in the dorsal skull, 2 mm anterior to the bregma and 1 mm lateral to the midline, for reference. Reference electrode (Nilaco, Tokyo, Japan; diameter: 0.13 mm; impedance <20 kΩ) was inserted into the hole and securely fixed at a depth of approximately 1 mm from the cortex surface with acrylic glue. Following the temporal fixation of electrodes with acrylic glue, the entire exposed skull was covered with dental resin (Super bond C&B; Sun Medical, Shiga, Japan). A V-shaped metal plate (F-911; Hilogik, Osaka, Japan) was attached to the dental resin over the skull using dental cement (Province; Shofu, Kyoto, Japan), allowing the subjects to be head-fixed during the experiment.

The left side of the pinna was detached to provide a clear view of the tympanic membrane and outer canal. An incision of the muscle and skin from the shoulder to the jaw exposed the left side of the bulla, and a 1-mm diameter hole was made on the bulla. A silver electrode for recording cochlear responses (Nilaco, Tokyo, Japan; diameter: 0.13 mm; impedance <20 kΩ) was inserted into the hole and secured onto the bony rim of the round window. The electrode hole was completely sealed using acrylic glue and dental cement (Provinice; Shofu, Kyoto, Japan) to stabilize the electrode with the bulla and preserve moisture in the middle ear. All incised muscles and skin were carefully sutured after providing a mixture ointment of colistin sulfate and bacitracin (dolmaisin ointment; Zeria, Tokyo, Japan). For postoperative analgesia, lidocaine solution (Xylocaine 0.5% for Intramuscular Injection; Aspen Japan, Tokyo, Japan) was applied locally to the surgical site twice daily for 2 days.

After surgery, all subjects were individually housed and given a recovery period of at least two days before training commenced. The animal surgery followed the same procedure as in our previous studies.^16^^,^^58^

#### Auditory stimuli

Click trains with a repetition rate of 4 kHz served as auditory stimuli. The click train comprised 0.1 ms rectangular pulses, and the duration of auditory stimulation was 500 ms. The auditory stimulus was delivered through a speaker (AS01008MR-2-R, Pui audio, Ohio, America), positioned 4.5 cm vertically downward at a 45° angle from the gerbil’s left ear. Auditory stimulus generation employed a digital-to-analog converter (Octa-Capture, Roland, Shizuoka, Japan) with a sampling rate of 192 kHz. Signal amplification was achieved using an amplifier (A-10, Pioneer, Tokyo, Japan). The sound pressure level was adjusted within the range of 20–95 dB SPL using a microphone (Type 7016, Aco, Tokyo, Japan).

#### Laser stimuli

A repetitive pulsed infrared laser was employed for the infrared laser stimuli. Each individual laser pulse had a rectangular shape in intensity, lasting 0.1 ms, and the inter-onset interval was 0.25 ms, corresponding to a 4-kHz repetition rate. The duration of the pulsed infrared laser train was 500 ms. The pulsed infrared laser was generated using a continuous mode diode laser stimulation system (BWF-OEM, B&W TEK, Delaware, USA) with a wavelength (λ=1871) consistent with previous studies.^16^^,^^17^^,^^20^^,^^34^^,^^58^^,^^59^ Voltage commands for the laser stimuli were generated using a digital-to-analog converter (Octa-Capture, Roland, Shizuoka, Japan). The laser was delivered through an optical fiber (diameter: 100 μm; NA: 0.22) inserted mediolaterally into the left outer ear canal, with the tip angled rostrally by 10° and dorsally by 5° using a micromanipulator (MM-3; Narishige, Tokyo, Japan) in awake head-fixed subjects. The optic fiber tip was fixed at approximately 0.7 mm (the distance between the cochlea and fiber tip was about 2.2 mm) in front of the tympanic membrane to ensure transtympanic irradiation of the cochlea from the outer ear, as described in our previous reports.^16^^,^^17^^,^^58^^,^^59^ The laser beam profile at the cochlea (2.2 mm from the fiber tip) was measured, as in previous studies.^16^^,^^58^ Radiant energy was measured using a digital power meter (PM100D; Thorlabs, Tokyo, Japan) with a thermal power sensor (S302C; Thorlabs, Tokyo, Japan), calibrated between 0.1 and 13.2 mJ/cm^2^. The beam width (full width at half the maximum diameter) at the cochlea was calculated using an InGaAs-biased detector (DET10D/M; Thorlabs, Tokyo, Japan) and measured as 2.2 mm.

#### Visual stimuli

A white light-emitting diode (LED) (diameter: 5.0 mm) was used for visual stimulation. The LED was placed 10 mm from the left eye of the gerbil in the horizontal plane. The voltage command for the visual stimulus was controlled using a digital-to-analog converter (Octa-Capture, Roland, Shizuoka, Japan) with a 192-kHz sampling rate. Light stimulation was applied for 500 ms, and light intensity was calibrated at 96 lux using a digital lux meter (GL-03; be-s, Osaka, Japan).

#### Apparatus used in the behavioral experiment

Behavioral experiments and electrophysiological recording were performed in the same 60 × 60 × 60-cm (W × L × H) soundproof box, following our previous study.^58^ Each subject was positioned on a custom-designed covered platform with a copper sheet. The subjects’ heads were stabilized by clamping the V-shaped metal plate attached to the heads. A metal drinking spout was fixed around 1 mm in front of the mouth of the subjects. A touch sensor (DCTS-10; Sankei Kizai, Tokyo, Japan), connected between the drinking spout and copper sheet of the stage, recorded the timing of licks at 2000 samples/s. Voltage changes from the touch sensor (licking timing) were captured by Arduino Uno (Arduino, Ivrea, Italy) using Python 3.7 on Spyder 501 and stored on a computer. All behaviors were recorded using a video camera (HD-5000; Microsoft, Washington, USA).

#### Procedure of the training and test session

The training with auditory, laser, or visual stimulation was conducted in separate experimental groups (Auditory: *n*=15; Laser: *n*=12, Visual: *n*=4). After at least two days of recovery from surgery, the gerbils underwent water restriction in their home cage. The training session typically consisted of 250–400 trials per day. The head-fixed animals could voluntarily lick the spout throughout the behavioral experiment. A 3.2 μL drop of water (unconditioned stimulus: US) was delivered through the tube after a short interval, 678 ± 32 ms (mean±SD, *n*=120), from the onset of auditory, laser, or visual stimulation (i.e., conditioned stimulus: CS). The inter-trial interval was randomly varied between 7 and 13 s. The timing of US and CS was controlled using MATLAB (MathWorks, Massachusetts, USA) and a microcontroller (Arduino Uno; Arduino, Ivrea, Italy) with a custom-made relay circuit.

After animals exhibited elevated licking rates following the CS without the delivery of the US, a test session was conducted. The test session consisted of 280 rewarded trials and 120 nonrewarded trials (i.e., catch trials). In rewarded trials, the CS and US were delivered as in the training session, aiming to sustain a heightened motivational state in the subjects. Catch trials evaluated the subjects’ perceptions without reward feedback. Six stimulus types were presented 20 times in catch trials, and the behavioral response to each stimulus was examined. Additional details for each stimulus condition are provided in Figure S1. Changes in licking behavior with conditioning are shown in Figure S2.

#### Recording and analysis of behavioral data

An averaged peri-stimulus time histogram (PSTH) of licking behavior assessed the time course of behavioral responses. PSTHs were calculated in a 100-ms bin size. To smooth the PSTHs, a three-point moving average (i.e., 300 ms window) was applied. To quantify behavioral response, peak licking rates, durations, peak latency, response latencies, and delta licking rate (DLR) were extracted from PSTHs, as in our previous study.^58^ Peak licking rates and latency were defined as the licking rate and the time when the licking rate reached its maximum value within 4 s after stimulus onset, respectively. The baseline licking rate was determined as the prestimulus licking rate obtained for 1 s, and duration was quantified as the temporal length of PSTH >50% of the licking rate from the mean baseline licking rate to the peak licking rate. Response latency was calculated by measuring the time between stimulus onset and the behavioral response reaching the mean plus three standard deviations of the baseline licking rate. The DLR was defined as the difference between the mean baseline licking rate and the post-stimulus licking rate (analysis range: 0.2–1.2 s) to evaluate the response amplitude. The analysis range of post-stimulus licking behavior was determined by latency data of the behavioral response (Figure S5) and our previous study.^58^

#### Electrophysiological recording during the behavioral experiment

The cochlear response during the behavioral experiment was recorded and amplified 1000 times using a bio-amplifier (MEG-1200; Nihon Kohden, Tokyo, Japan). Electrophysiological data were stored on a computer using an analog-to-digital converter (Octa-capture; Roland, Shizuoka, Japan) at a sampling rate of 96 kHz.

The recorded signal underwent processing with a band-pass filter, employing 1024 digital sampling points (500–3500 Hz) to minimize summating potentials and CMs while extracting the CAP of the cochlear nerves. CAP amplitude was defined as the peak-to-peak voltage between the first minimum (N1) and maximum (P1) of each CAP response.

In the auditory masking experiment, defining the peak of CAP became challenging due to the substantial CM elicited by loud white noise (i.e., masker) compared to CAP. The band-pass filter could not entirely eliminate CM. Consequently, the change in the root-mean-square (RMS) amplitude between pre- and post-signal onset was measured (i.e., diff RMS amplitude). Signal onset was determined through visual inspection using MATLAB (MathWorks, Massachusetts, USA). Pre- and post-CAP signals for 2 ms from signal onset were obtained to calculate the relative voltage change in CAP triggered by auditory and laser stimulation.

### Quantification and statistical analysis

#### Data analysis

Statistical analyses were performed using MATLAB, Python, and R. Data are presented as mean ± SD or SEM, as indicated in each figure. Sample sizes (n) are specified in the corresponding figure legends. To assess relationships among variables, Pearson’s correlation analysis and linear regression analysis were conducted. ANCOVA was used to evaluate differences in the slopes and intercepts of linear regression models between physiological and behavioral responses across conditions. For comparisons across multiple stimulus conditions, two-tailed paired t-tests with Bonferroni correction were applied where appropriate. Statistical significance was defined as *p* < 0.05. Significance levels are indicated as follows: ∗*p* < 0.05, ∗∗*p* < 0.01, and ∗∗∗*p* < 0.001.
