# Supplementary material for: Optical induction of auditory perception via cochlear stimulation in Mongolian gerbils without genetic modification
Source: iScience. 2026 Jun 30;29(7):116588. doi: 10.1016/j.isci.2026.116588 (PMC13378388; doi:10.1016/j.isci.2026.116588)
Supplement: Document S1. Figures S1–S10 [file mmc1.pdf]

iScience, Volume *29*

Supplemental information

Optical induction of auditory perception via cochlear stimulation in Mongolian gerbils without genetic modification

Yuta Tamai, Miku Uenaka, Aya Okamoto, Yuki Ito, Kaito Fukada, Ayase Kawasaki, Takaki Shintani, Riko Nakagawa, Koji Toda, Shizuko Hiryu, and Kohta I. Kobayasi

Supplemental figure

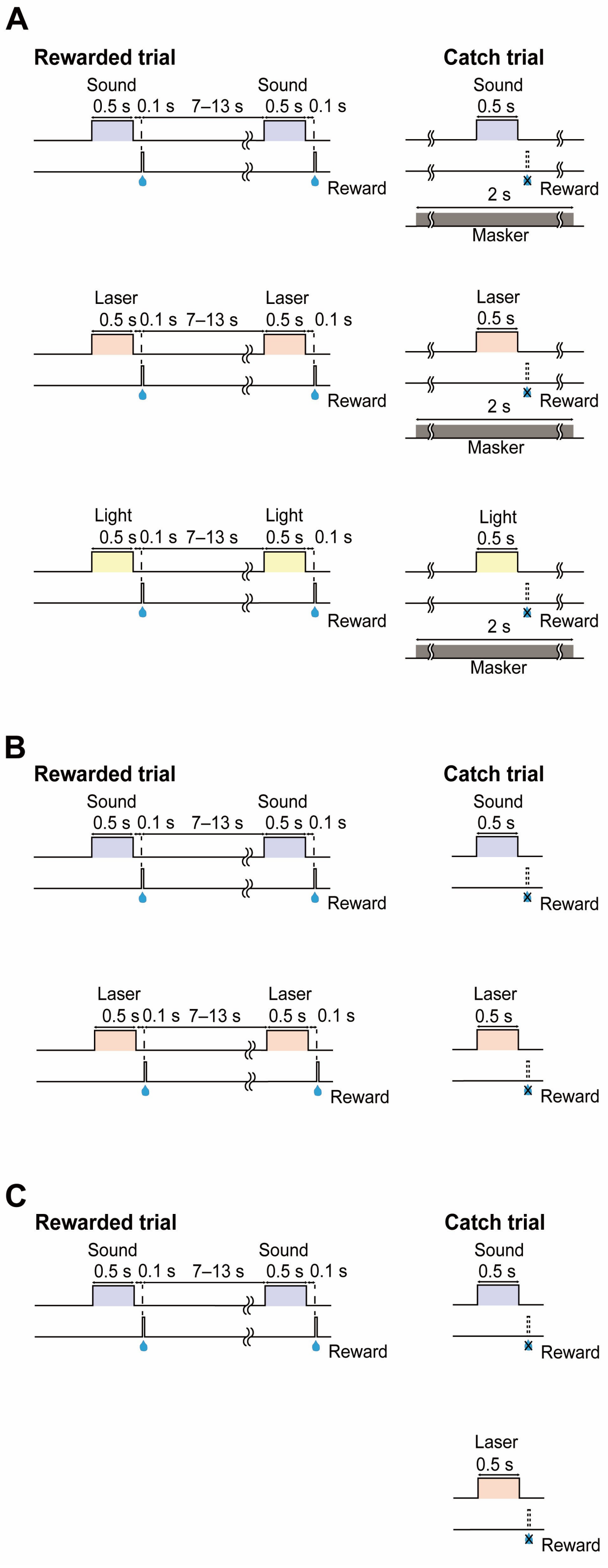

Fig. S1. Schematic of conditioned (CS) and unconditioned (US) stimulus presentation in the mono-stimulus experiment. The behavioral task of effect of auditory masking on auditory- and laser-evoked responses (A), intensity dependance of auditory- and laser-evoked responses (B), and assessment of stimulus generalization (C) are shown. The timing of CS and US in the rewarded (left) and catch trials (right) are described. In (A), 15 auditory (70 dB SPL)- and 6 laser (11.7 mJ/cm^2)-trained animals were used. In the rewarded trial (left), click train (70 dB SPL) or pulsed laser train (11.7 mJ/cm^2) was presented to the auditory- and laser-trained animals, respectively. In the catch trials (right), the sound pressure level and radiant energy of CS were varied (auditory: 20, 35, 50, 65, 80, or 95 dB SPL; laser: 0.1, 1.1, 3.1, 6.9, 10.2, or 13.2 mJ/cm^2). In (B), the experiment used six auditory- (80 dB SPL), six laser- (13.2 mJ/cm^2), and four visual- (96 lux) trained animals. In the rewarded trial, click train (80 dB SPL), pulsed laser train (13.2 mJ/cm^2), or LED light (96 lux) was presented to the auditory-, laser-, or visual-trained animals, respectively. To investigate the effect of auditory masking on the physiological and behavioral responses induced by auditory, laser, and visual stimulation, white noise (700–44,800 Hz) of 0, 35, 50, 65, 80, or 95 dB SPL for 2 s was presented during the auditory, laser, or visual stimulus periods of catch trials. In (C), all animals (n=11) were trained by auditory (70 dB SPL) stimulation. In the rewarded trials, a click train was used. Auditory (20, 50, and 80 dB SPL) and laser (0.1, 6.6, and 13.2 mJ/cm^2) stimuli were introduced in the catch trials.

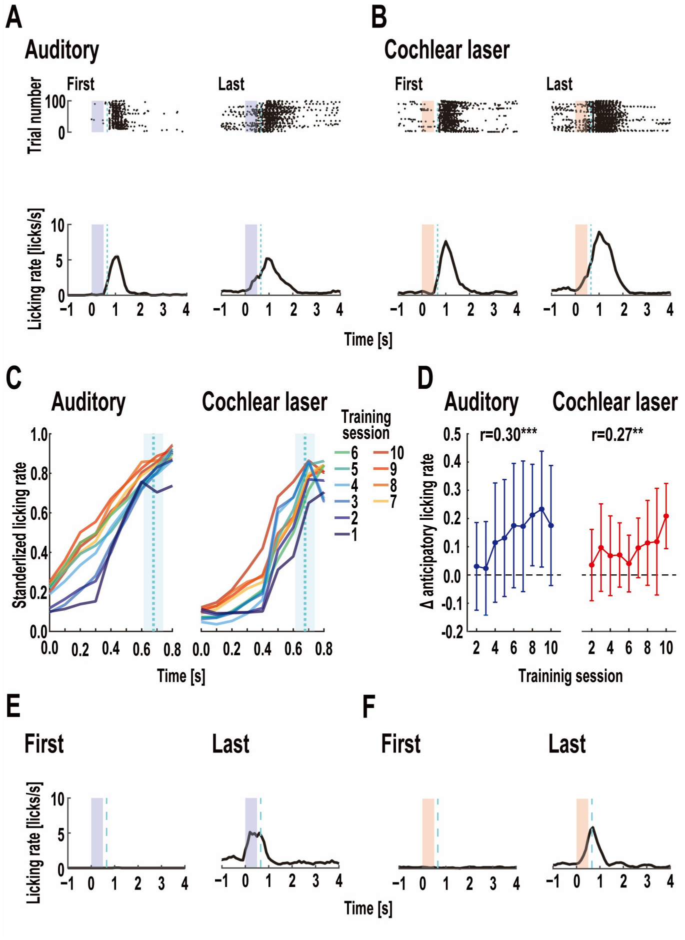

Fig. S2. Licking behavior transition by Pavlovian conditioning with auditory and laser stimulation. Auditory- (A) and laser-induced (B) licking behavior in the first (1–100 trials) and last (901–1000 trials) training session. Individual trials (top) and mean peri-stimulus time histogram (PSTH) of licking behavior (bottom) elicited by auditory and laser stimulation are described. The blue and red zones indicate the auditory and laser stimulation periods, respectively. The blue dashed lines indicate the reward timing. The black plots in the top figure show the licking timing. In the first training session, auditory- and laser-evoked licking responses were observed after US presentation, while these licking behaviors were also recorded before US presentation in the last training session. (C) Changes in average auditory (n=13)- and laser (n=12)-CR in each training session. One training session comprised 100 trials. The blue dashed lines and areas show the mean reward timing and 2SD. The standardized licking rates until 0.6 s after stimulus onset were measured as CR. The gradual increase in CR amplitude was observed every session. (D) Change in the Δ anticipatory licking rate within the CS-US interval (0–0.6 s) across

training sessions. Delta anticipatory licking rate was calculated by subtracting the average licking rate during the CS-US interval in the first session from that in each subsequent session. The delta anticipatory licking rate significantly increased as training progressed (Auditory: r=-0.30, $P<0.001$; Laser: r=-0.27, $P<0.01$; Pearson's correlation analysis). ANCOVA showed no significant difference in slopes ($F(1, 221)=1.178$, $P=0.279$; ANCOVA), but a significant difference was observed in y-axis intercepts ($F(1,222)=4.221$, $P=0.041$; ANCOVA). Error bars show SDs. The Auditory- (E) and laser-induced (F) average PSTH of licking rates on the first and last day of catch trials (1–120 trials). The blue and red zones indicate the auditory and laser stimulation periods, respectively. The blue dashed lines indicate the reward timing in the rewarded trial. Catch trials were performed to monitor the association between CS and US. Synchronous increase in licking rates with auditory and laser stimulation were not obtained on the first day of the catch trial. After completing the training, synchronous licking behaviors with CS were observed.

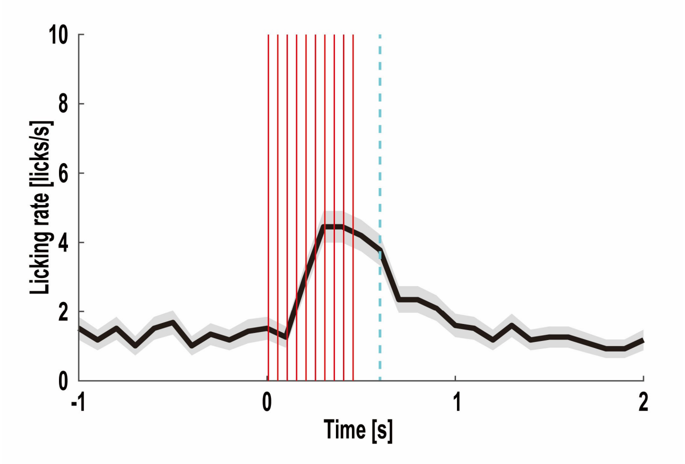

Fig. S3. Licking behavior elicited by a 20 Hz pulsed laser train (i.e., an inter-pulse interval of 50 ms) delivered for 500 ms. An increase in licking rate became apparent after the initial 100-ms bin following stimulus onset. Because the PSTH was calculated in 100-ms bins and smoothed using a 300-ms sliding window, the apparent latency may be slightly influenced by the smoothing procedure. Nevertheless, these results suggest that laser-induced perception may arise with only a small number of laser pulses (one or two pulses). Red lines show the timing of each laser pulse. The light blue dotted line indicates the reward timing during the training session. Gray areas show the SEM across trials.

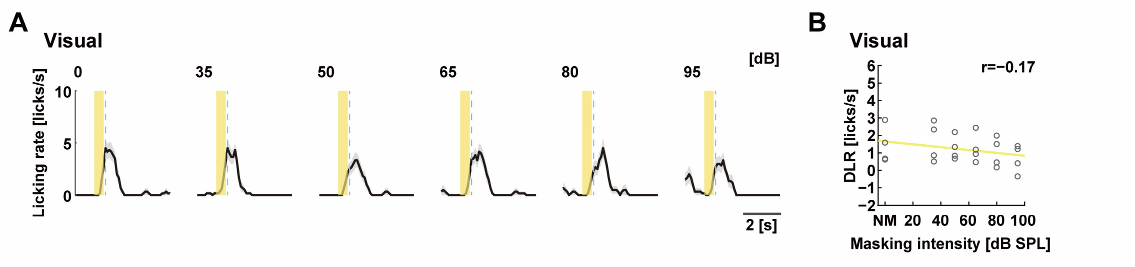

Fig. S4. Effect of auditory masking on visually conditioned response. This investigation was performed as a control condition to ensure that the presence of intensive white noise did not cause behavioral disturbance or interfere with auditory input. (A) Mean PSTH of licking behavior elicited by visual stimulation in the masking experiment. Yellow areas indicate stimulus duration. Blue dashed lines indicate reward timing in a training session. (B) Correlation between masking intensity and visually evoked DLR. The result of the control experiment showed that a significant decrease in visual-evoked DLR was not observed as the sound pressure level of white noise increased (r=-0.17, *P*=0.310; Pearson’s correlation analysis); therefore, auditory masking in this experiment mainly interrupted auditory-related perception.

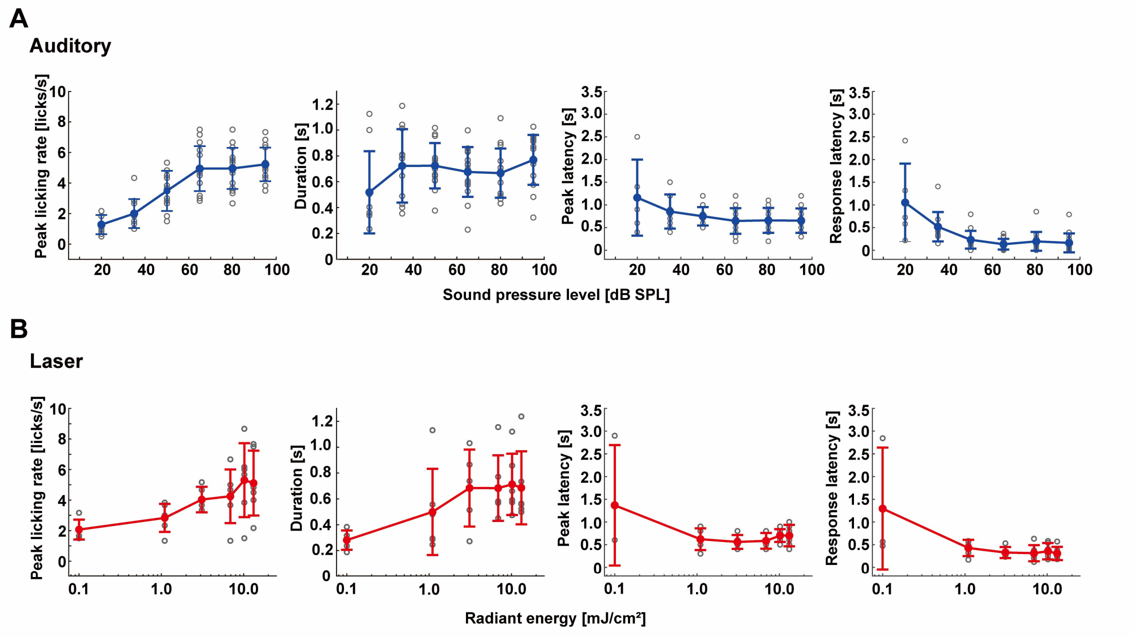

Fig. S5. Changes in features of behavioral responses by modulating sound pressure level and radiant energy. Intensity dependance of the peak licking rate (leftmost), duration (left), peak latency (right), and response latency (rightmost) with auditory (A) and laser (B) stimulation. Error bars show the standard deviations. As the sound pressure level increased from 20 to 95 dB SPL, the peak licking rate rose from 1.28±0.63 to 5.22±1.10 licks/s; duration increased from 0.52±0.32 to 0.77±0.19 s; peak latency decreased from 1.16±0.84 to 0.65±0.27 s; and response latency decreased from 1.05±0.86 to 0.16±0.21 s. As radiant energy increased from 0.1 to 13.2 mJ/cm^2, the peak licking rate rose from 2.07±0.65 to 5.11±2.13 licks/s; duration increased from 0.28±0.07 to 0.69±0.28 s; peak latency decreased from 1.37±1.33 to 0.70±0.24 s; and response latency decreased from 1.29±1.34 to 0.31±0.15 s. The properties of auditory-evoked behavioral responses were similar to those in previous studies [5] and to those of laser-evoked behavioral responses.

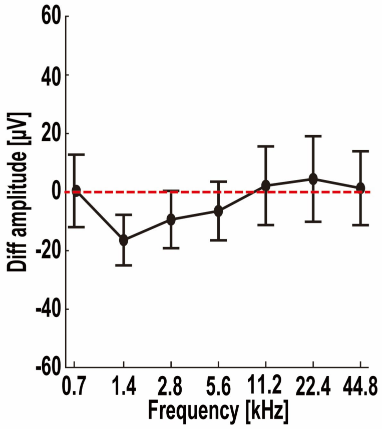

Fig. S6. Tone masking tuning curve of laser-evoked cochlear response. Subjects were anesthetized using an intramuscular combination injection of ketamine (47.0 mg/kg) and xylazine (9.3 mg/kg), and cochlear responses were recorded from the round window of the cochlea with a silver electrode. The tuning curve exhibited a best frequency of 1.4 kHz, with masking effects observed at 2.8 and 5.6 kHz. Diff amplitude represents the difference in the amplitude of laser-evoked cochlear responses before and after masker presentation; a value of 0 indicates no change in response amplitude. Error bars indicate SEMs.

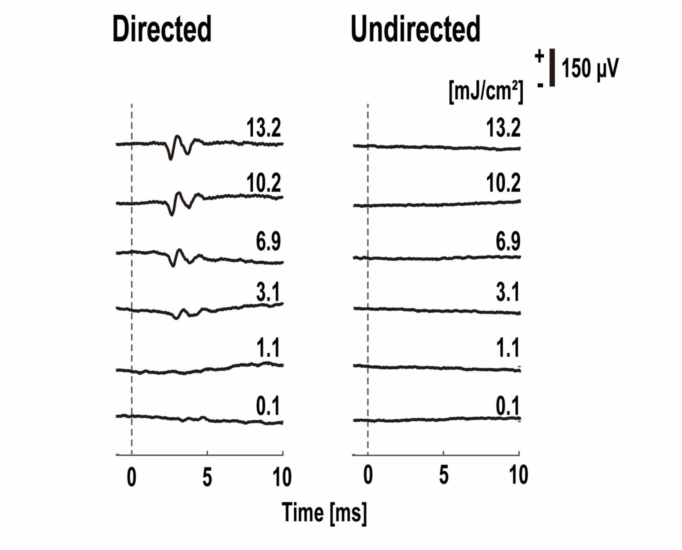

Fig. S7. Cochlear response induced by the directed and undirected laser stimulation from the entrance of the ear canal. Directed laser stimulation was delivered from the laser fiber placed at the entrance of the ear canal to the cochlea through the tympanic membrane. Undirected laser stimulation was defined as orienting the laser fiber ventrally and positioning the fiber tip at the entrance of the ear canal, from which the laser irradiation was applied. A 4 kHz pulsed laser train of 500 ms duration, identical to that used in the behavioral experiment, was presented. Dotted indicates stimulus onset.

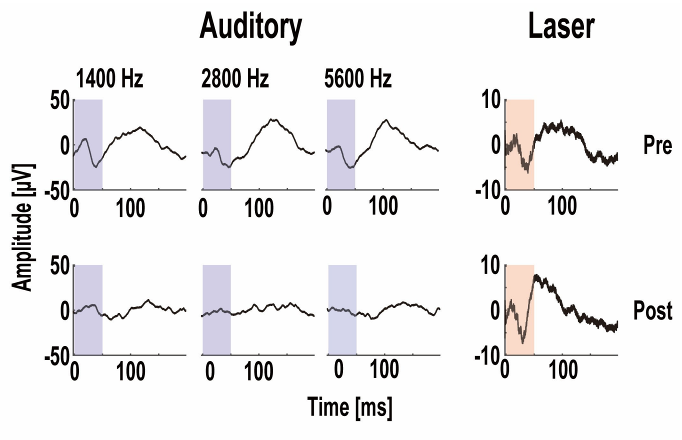

Fig. S8. Auditory evoked potential (AEPs) induced by auditory and laser stimulation pre- and post-neomycin administration. AEPs were recorded from the auditory cortex using a silver electrode in anesthetized subjects. Anesthetization was induced by an intramuscular combination injection of ketamine (47.0 mg/kg) and xylazine (9.3 mg/kg). Neomycin (200 µl , 200 mM) was applied to the round window of the cochlea, and AEPs were recorded before and after administration. Auditory stimuli consisted of 80 dB SPL tone bursts at 1400, 2800, and 5600 Hz, which fall within the frequency range in which tone masking of laser stimulation is effective (Fig. S6). Laser stimulation consisted of a repetitive pulsed laser train (inter-pulse interval: 8 ms) at 3.1 mJ/cm^2. The duration of auditory and laser stimulation was 500 ms. The blue and red areas indicate the auditory and laser stimulation periods, respectively. After neomycin administration, AEPs induced by auditory stimulation were markedly reduced, whereas those AEPs elicited by laser stimulation were preserved.

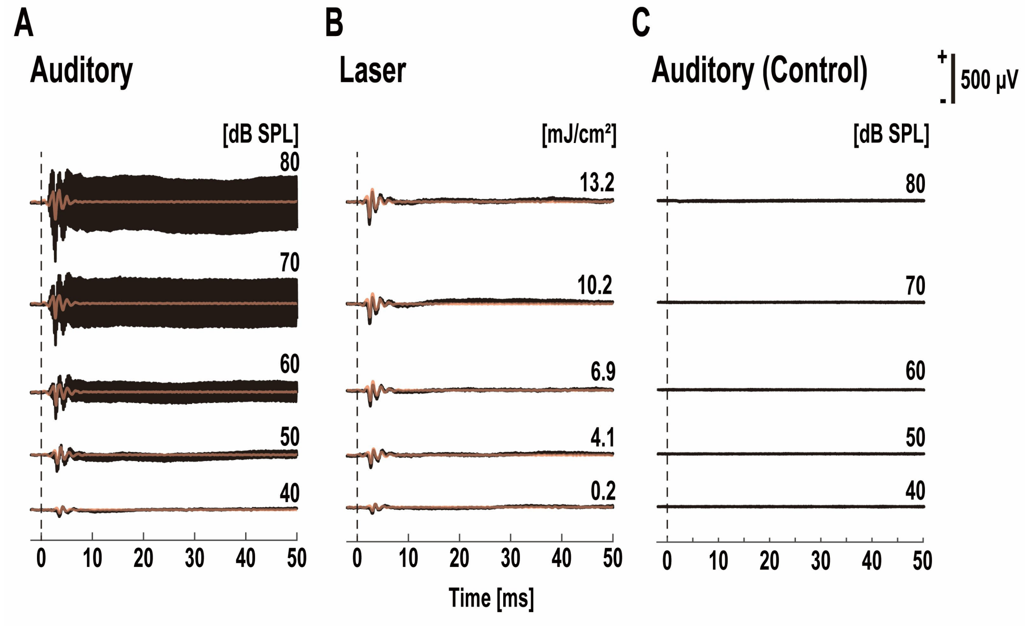

Fig. S9. Intensity dependence of the cochlear response before and after euthanasia. Cochlear response was recorded from round window of the cochlea with a silver electrode in anesthetized subjects, using the same method as described in Fig. S6. Auditory (A)- and laser (B)-induced cochlear response with (red) and without (black) low-pass filter prior to euthanizing the animals. (C) Auditory-induced cochlear response recorded after euthanasia. The response was recorded 1h after euthanasia with an overdose of pentobarbital (200 mg/kg i.p.). The subject's heartbeat and respiration had ceased within 1 h after the pharmacological intervention. The same stimulus parameters (i.e., stimulus duration, repetition rate of clicking sound, and sound pressure ranges) were used. Dotted lines show stimulus onset. The post-euthanasia recordings were performed to confirm that the sound-evoked cochlear responses were physiologically generated rather than electrical artifacts from the acoustic stimulation system.

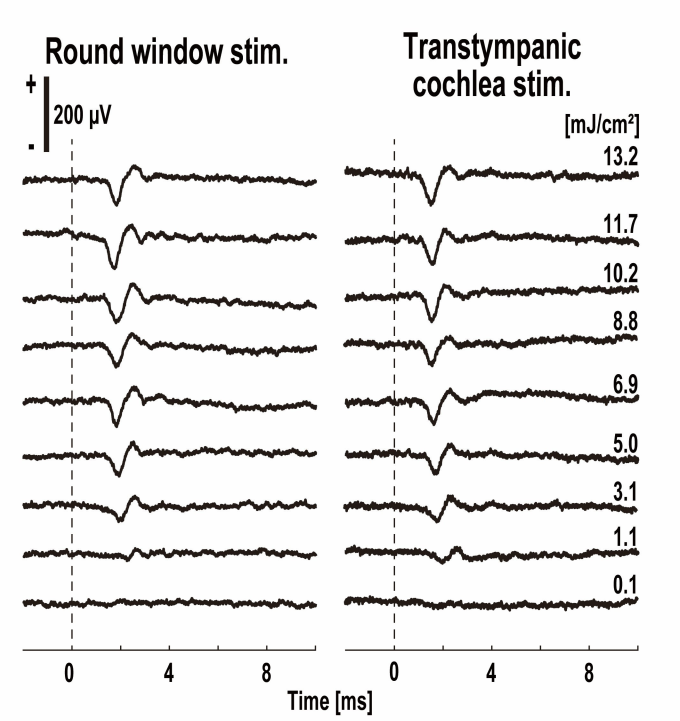

Fig. S10. Cochlear response elicited by round window stimulation and transtympanic laser stimulation. Cochlear response was recorded from the round window of the cochlea with a silver electrode in an anesthetized subject, following the method described in Fig. S6. This result shows that cochlear responses elicited by transtympanic laser stimulation were comparable to those elicited by round window laser stimulation, which directly irradiated the cochlea without requiring penetration of the tympanic membrane. Data are shown from a representative subject (n=1).
